# Czech Women’s Point of Views on Immediate Breast Reconstruction after Mastectomy due to BRCA Gene Mutation or Breast Cancer

**DOI:** 10.3390/healthcare11121755

**Published:** 2023-06-15

**Authors:** Tomáš Ventruba, Michal Ješeta, Luboš Minář, Jindřich Vomela, Dagmar Brančíková, Jana Žáková, Pavel Ventruba

**Affiliations:** 1 Department of Obstetrics and Gynecology, University Hospital Brno and Masaryk University, 625 00 Brno, Czech Republic; 2 V-CLINIC, Clinic of Plastic, Aesthetic Surgery and Gynecology, 603 00 Brno, Czech Republic; 3 Department of Surgery, University Hospital Brno and Masaryk University, 625 00 Brno, Czech Republic; 4 Department of Internal Medicine, Hematology and Oncology, University Hospital Brno and Masaryk University, 625 00 Brno, Czech Republic

**Keywords:** immediate breast reconstruction, mastectomy, BRCA mutations, breast cancer, patient awareness, questionnaire study

## Abstract

(1) Objective: Breast cancer is the most common cancer in women, and the incidence of the disease continues to increase. The issue of immediate breast reconstruction (IBR) in women with BRCA mutations and breast cancer is highly topical. This study is based on the long-term experience of our workplace with the diagnosis and treatment of breast cancer in women. We use the possibilities of oncoplastic surgery, including IBR. Our effort involves learning about women’s awareness of IBR with a mastectomy at the same time. (2) Methods: The method of quantitative research of women’s awareness using a structured anonymous questionnaire was chosen. Out of the total number of 84 respondents who already underwent IBR, 36.9% were due to BRCA mutations, and 63.1% were due to breast cancer. (3) Results: All of the respondents learned about the possibility of IBR before treatment or during treatment planning. The information was first obtained mainly from an oncologist. Women obtained the most information regarding IBR from a plastic surgeon. Before the mastectomy, all of the respondents already knew what IBR meant, as well as about the payment of IBR by the health insurance company. All of the respondents would choose the IBR option again. A total of 94.0% of women cited preservation of body integrity as a reason for undergoing IBR, and 88.1% of women knew about the possibility of performing IBR with their own tissues. (4) Conclusions: There are few specialized centers with a team of experts in reconstructive breast surgery in the Czech Republic, especially those that perform IBR. Research has shown that all of the patients were well informed about IBR, but the vast majority only learned about IBR before the surgical procedure was planned. All of the women wished to maintain body integrity. Our study results in the recommendations for patients and for healthcare management.

## 1. Introduction

Breast cancer is the most common cancer in women and one of the most common causes of death worldwide [1]. The incidence of the disease continues to increase, while the mortality rate, on the other hand, has been stagnant since the mid-1990s and has slightly decreased. In 2020, breast cancer became the leading type of malignancy worldwide, with 2.3 million new cases per year [2]. The incidence rates are the highest in Australia, New Zealand, North America, and western and northern Europe. This is thought to be related to a higher prevalence of reproductive and hormonal risk factors (e.g., later age at menopause and fewer children), lifestyle risk factors (i.e., alcohol intake, excess body weight, and physical inactivity), and increased detection through mammographic screening [2,3].

In the Czech Republic, the incidence of breast cancer has increased in the past fifteen years from 122 to 138 cases per 100,000 women, or around 7700 new cases per year, of which more than 10% are in women under the age of 44 years [4,5]. With the ever-increasing incidence of breast cancer and stagnant or slightly decreasing mortality from it, an increase in prevalence, i.e., the number of living women who were diagnosed and treated for breast cancer in the past, is an inevitable consequence. The increasing incidence of breast cancer and its decreasing mortality lead to an increase in the number of women living with the long-term consequences of the surgical treatment of this disease [6]. In the context of the success of the treatment, the consequences of the therapy, which reduce the quality of life of the cured women, gain importance [7,8].

An inseparable part of the complex therapy of breast cancer is the surgical treatment. It requires a multidisciplinary approach that includes the participation of the surgeon, gynecologist, oncologist, and radiotherapist in cooperation with the plastic surgeon, geneticist, endocrinologist, and clinical psychologist. When deciding on the extent of surgical treatment, the patient’s age, the stage of the disease, and the patient’s individual preferences must be taken into account.

Women with mutations in the tumor suppressor genes BRCA 1 and BRCA 2 (breast cancer) have a significantly increased risk of developing breast cancer, which is the most common cancer in women, compared to the general population. Approximately 5 to 10% of all breast cancer cases in the Czech Republic, and 25 to 40% of cases diagnosed under the age of 35 years, have hereditary origins [9]. Tumors that occur at a younger age tend to be bilateral and multifocal. The BRCA 1 gene is located on chromosome 17, and the BRCA 2 gene is located on chromosome 13. The lifetime risk of breast cancer development in a woman with a congenital BRCA 1 mutation is 85%, and for a BRCA 2 mutation, it reaches 60–80%, against a cumulative 8–10% risk in the population [10,11]. Occurrence mutations in the BRCA1 and BRCA2 genes in the Czech Republic can be estimated at 2.8% based on the occurrence of population-specific variants in an unselected population of women with breast cancer [12].

The most effective prevention of breast cancer in BRCA mutations with a high genetic risk is the complete surgical removal of the mammary gland. A prophylactic mastectomy is mainly performed within the concept of immediate reconstruction. The postponement of the reconstruction is related to an unclear finding, which requires a definitive histological examination. With the development of genetic testing programs, the need for prophylactic procedures in mutated patients, as well as healthy carriers of the BRCA 1 and BRCA 2 tumor suppressor gene mutations, is increasing [13,14]. Newer methods also involve a combination of a curative procedure on one breast, and a simultaneous prophylactic procedure on the other breast [15].

Oncoplastic surgery has expanded the possibilities of breast oncological surgery, allowing for procedures that are sufficiently radical and with a very good cosmetic effect. In the early stages of breast tumors, breast oncological surgery even makes it possible to avoid chest radiotherapy, which, in the case of partial resections, is necessary to achieve sufficient local control of the tumor [16,17].

Breast reconstruction is the surgical creation of a missing breast using one’s own tissue or foreign material. From an oncological point of view, we cannot talk about a curative effect, but breast reconstruction can contribute to the improvement in the quality of life [18,19]. Breast reconstruction has undergone great development since the introduction of silicone implants into practice in 1963 [20]. Autologous tissue, foreign material, or their combination are used. The radicality of the mastectomy is decisive for the resulting aesthetic effect. The less radical the primary surgical procedure, the more effective the postoperative result of the breast reconstruction [21,22].

In Central Europe, including the Czech Republic, the financial limits of medical facilities are among the main obstacles that stand in the way of providing immediate and delayed breast reconstruction during the treatment of a patient with breast cancer. This is related to the fact that the most modern procedures are significantly underestimated by health insurance companies. The lack of funds and inflexible systems are the main reasons why the treatment results in Central Europe are not at a higher level than in other developed countries. The waiting times for oncoplastic procedures are still extremely long and very difficult to access in the countries of the Visegrad Group (Czech Republic, Hungary, Poland, and Slovakia—V4), especially in workplaces that are busy [23].

This work builds on our workplace’s long-term experience with the diagnosis and treatment of breast cancer in women. We use the possibilities of oncoplastic surgery, including immediate breast reconstruction at the same time as subcutaneous and skin-sparing mastectomy [24].

The aim of the research questionnaire is to learn about the opinions and information about immediate breast reconstruction (IBR) with mastectomy at the same time among women who already underwent these operations. The indication was a proven genetic mutation of BRCA 1 and 2 and/or breast cancer. The research was conducted at the Department of Gynecology and Obstetrics of Masaryk University (GPK MU) and University Hospital Brno, where IBR is performed at the same time.

A quantitative research method was chosen, and four sub-goals with hypotheses were set for two variables (n1, n2). The first group (n1) included patients with BRCA 1 and 2 mutations, in whom the risk of developing breast cancer is much higher than in the rest of the population. The second group (n2) included women with breast cancer. We focused on women who are dealing with this insidious disease, but also on those who could be affected by breast cancer due to their genetic anamnesis [24,25].

## 2. Materials and Methods

### 2.1. Treatment Procedure for Women with BRCA Mutations or Breast Cancer

The treatment procedure for carriers of BRCA mutation depends on the stage of detection of the genetic mutation. In case of an early detection, before the development of a malignant disease, during the screening examination of family anamnesis, the women are dispensed and are recommended the prophylactic bilateral mastectomy. Recommendation of immediate or postponed prophylactic adnexectomy up to the age of 45 years is related to the patient’s reproductive plan. Method of choice is the skin-saving mastectomy with immediate breast reconstruction at the same time. In women with BRCA 1, 2 mutation diagnosed after the diagnostics of breast carcinoma, the primary therapeutical procedure depends on the stage of tumor according to general recommendations.

Patients with early breast carcinoma after a breast-saving operation and after termination of adjuvant chemotherapy and radiotherapy are indicated for the bilateral breast ablation and immediate reconstruction. In advanced carcinoma, the non-adjuvant therapy is followed by mastectomy with an appropriate procedure in maxilla according to the primary finding and the effect of the non-adjuvant therapy, i.e., either biopsy of the sentinel lymph node or systematic dissection of axilla in the area of the first two levels. Reconstructive operation with prophylactic ablation of the other breast is not performed in one procedure. Non-adjuvant chemotherapy does not increase the risks of the reconstructive surgeries.

### 2.2. Technical Possibilities of Breast Reconstruction

- Silicone implant;
- Tissue expander + silicone implant;
- Local flap + silicone implant;
- Reconstruction using own fat tissue (lipofilling);
- Distant pedunculated flap (latissimus dorsi flap);
- Free flaps via microsurgical transfer.

Subsequent adjuvant radiotherapy could increase the number of complications. Thirty-nine studies involving 1,191,371 participants were studied. Patients who received left-sided radiotherapy, compared with those receiving right-sided radiotherapy, experienced increased risks of developing coronary heart disease (RR 1.29, 95% CI 1.13–1.48), cardiac death (RR 1.22, 95% CI 1.08–1.37), and death from any cause (RR 1.05, 95% CI 1.01–1.10). In a comparison of patients with radiotherapy and without radiotherapy, the RRs were 1.30 (95% CI 1.13–1.49) for coronary heart disease and 1.38 (95% CI 1.18–1.62) for cardiac mortality [26]. If the patient is indicated for adjuvant radiotherapy, we can also consider delayed reconstruction. We use the possibility of immediate breast reconstruction at the same time as subcutaneous and skin-saving mastectomy and modified mastectomy depending on the size and location of the tumor. We solve the reconstruction with an expander, and in the second time, by inserting a silicone implant, or directly by inserting the implant alone or in combination with the use of autologous tissue, depending on how further oncological treatment (chemotherapy or radiotherapy) is planned.

### 2.3. Questionnaire Study Design and Data Collection

The main goal of the research questionnaire was to learn about the opinions and information about immediate breast reconstruction with mastectomy among women who already underwent these operations and in patients with a proven BRCA 1 and 2 genetic mutation or with breast cancer. The method of quantitative research of women’s awareness using a structured anonymous questionnaire (20 polytomy questions in Czech language) was chosen (English version in Supplementary File S1). The questionnaire was approved by the Department of Organizational, Legal Affairs and Human Resources of the University Hospital Brno (16.5.2019—2019/74695/FN Brno-1719).

The questionnaire survey was conducted at the plastic and reconstructive surgery clinic of the GPK MU Brno in the period from May 2019 to February 2020. A total of 84 female respondents were approached during the postoperative check-ups, all of whom filled out the questionnaire (return rate 100%).

The first two demographic questions concern the distribution of female respondents according to the type of tumor for which they underwent immediate reconstruction, and their age. Questions 3–8, 12 were focused on the patients’ awareness before the operation, satisfaction with the performance after the operation, and how they learned about the possibility of reconstruction. Satisfaction with the performed operation was summarized in questions 9–11, 16. Questions 13–15 related to knowledge of silicone implants. Questions 17–20 asked for information about postoperative risks.

## 3. Results

### 3.1. Oncoplastic Surgery Program

One hundred and three reconstructive surgeries were performed on 58 women with breast cancer or BRCA mutation carriers at GPK MU Brno in the period of April 2017–May 2020. In the subset of IBR, 52 procedures (50.5% of reconstructive surgeries) were performed in 48 women (82.8% of the operant group) with non-advanced breast cancer or BRCA1/2 mutation. The mean age was 48.2 ± 9.6 years.

**Figure 1:**
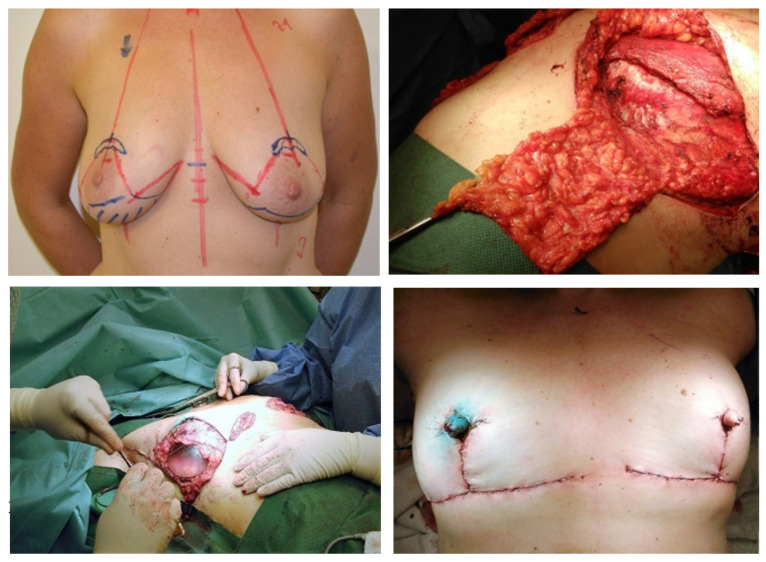
Immediate breast reconstruction in a patient with BRCA1/2 mutation and right breast tumor. Combined procedure was performed, including skin-saving mastectomy with immediate re-construction with implants, and creation of non-nipples using a local lobe and laparoscopic removal of the ovaries.

A tissue expander was inserted in 27 women (46.6% of the group) with locally advanced tumors and the need for subsequent radiotherapy (18 immediate and 9 delayed reconstructions) (Figure 1). Breast implants were used in 52 women (89.7% of the group), with a total of 80 implants. Breast reconstruction using the patient’s own tissues was performed in eight women, of which five operations were in the immediate reconstructiongroup. Postoperative complications occurred in 11 women, and 15 corrective procedures were performed (12.7% of operations).

### 3.2. Questionnaire Survey

**Figure 2:**
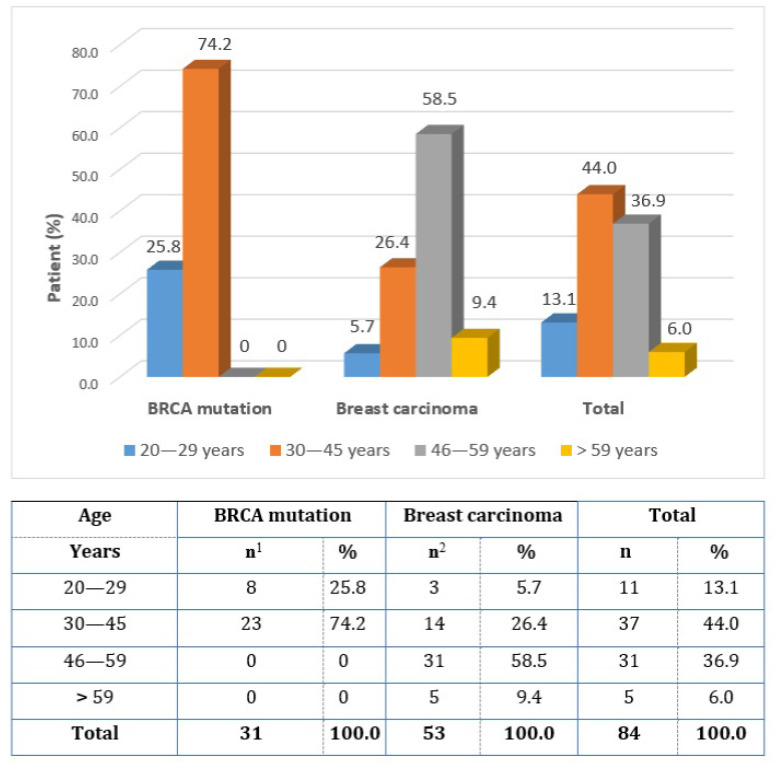
The reason for immediate breast reconstruction and the age structure of the respondents.

Out of the total number of 84 respondents, 31 women (36.9%) underwent IBR due to BRCA 1, 2 mutations, and 53 women (63.1%) underwent IBR due to breast cancer (question 1). The 30–45-year-old age group (39.2 ± 8.4 years) predominated in women with the BRCA mutation, compared to the predominant 46–59-year-old age group with breast cancer (49.6 ± 9.8 years). The age distribution of the group was assessed via question 2, and is shown in Figure 2.

**Figure 3:**
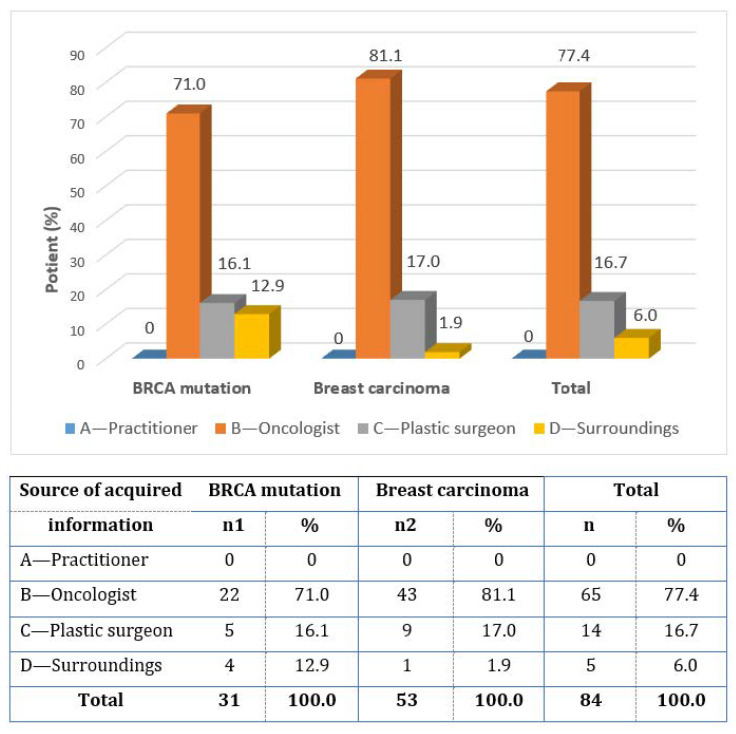
The first information obtained about immediate breast reconstruction.

All of the respondents learned about the possibility of IBR (question 3) before treatment (6.0%) or during treatment planning (94.0%). The information was first obtained mainly from an oncologist (77.4% of women)—question 4 (Figure 3). The answers to question 5 show that women obtain the most information regarding reconstruction surgery from a plastic surgeon (91.7%, respectively 83.9% in women with a BRCA mutation and 96.2% in women with breast cancer). Only 8.3% of women received information from their surroundings and from the Internet.

Before the mastectomy, all of the respondents already knew what IBR meant (question 6). The answer YES was given by 94.0% of women, and RATHER YES by only 6.0% of women, and the answers NO and I DON’T KNOW were not given by any of them. Information about the payment of the immediate reconstruction by the health insurance company was equally good (question 7). Only 7.2% of the female respondents said I DON’T KNOW, with the remaining 92.8% being women who knew about the payment and said YES. No difference in the BRCA mutation and breast cancer group was found.

**Figure 4:**
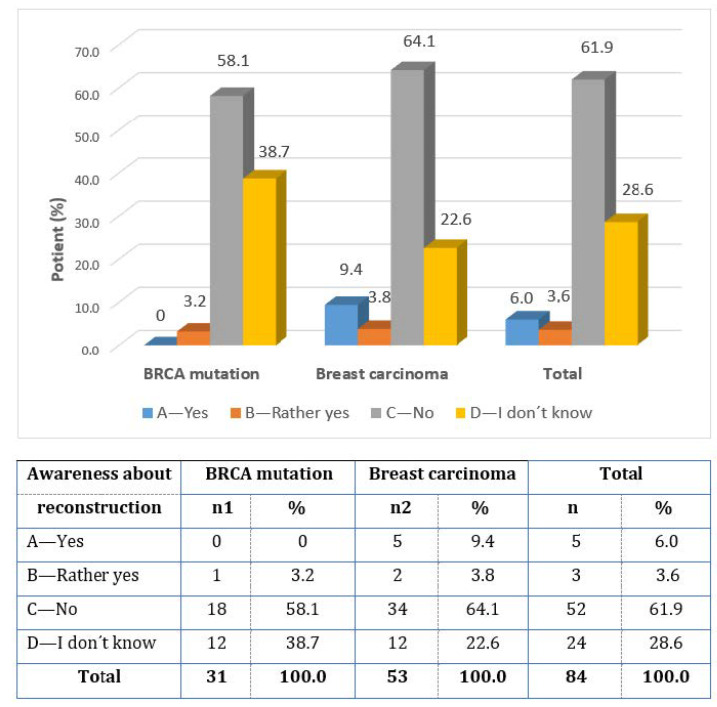
Information about immediate breast reconstruction in the Czech Republic.

When asked whether there is sufficient information about IBR in the Czech Republic (question 8), 61.9% of women answered ”NO”, and 28.6% of women answered ”I DON’T KNOW” (Figure 4).

All of the respondents would choose the IBR option again (question 9). In question 10, 94.0% of women cited preservation of body integrity as a reason for choosing IBR (all with BRCA mutation and 90.6% with breast cancer), and 9.4% of women with breast cancer answered I DON’T KNOW. No patients chose the reason “surroundings perception”. All 84 operatives would also recommend immediate breast reconstruction to women with the same diagnosis (question 11).

According to question 12, surprisingly, 88.1% of women (all 31 operant women for BRCA mutations and 81.1% with breast cancer) knew about the possibility of IBR using their own tissue. Only 8.3% of female respondents answered I DON’T KNOW, and 3.6% answered “I WASN’T INTERESTED”, when all of them were from the group of women with breast cancer. There was also excellent awareness of IBR via foreign material, always 100% in both groups (question 13).

**Figure 5:**
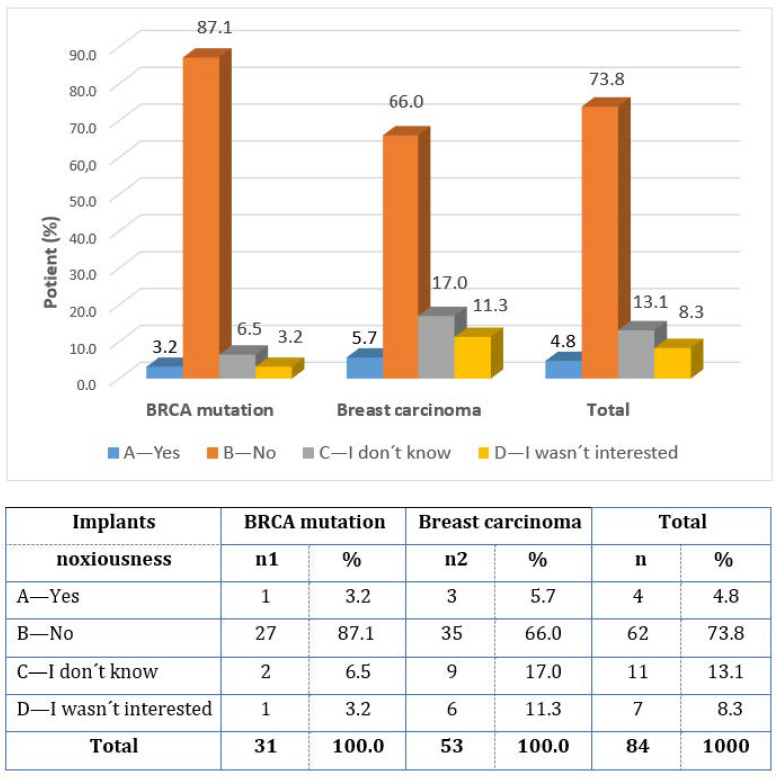
Opinion on the harmfulness of silicone implants.

There were differing views on the harmfulness of silicone implants (question 14). Only 4.8% of women said “YES”, while 73.8% of patients answered “NO”, and 13.1% of patients answered “I DON’T KNOW”. The responses of women with BRCA mutations differed fundamentally from the responses of operant women for breast cancer (Figure 5).

**Figure 6:**
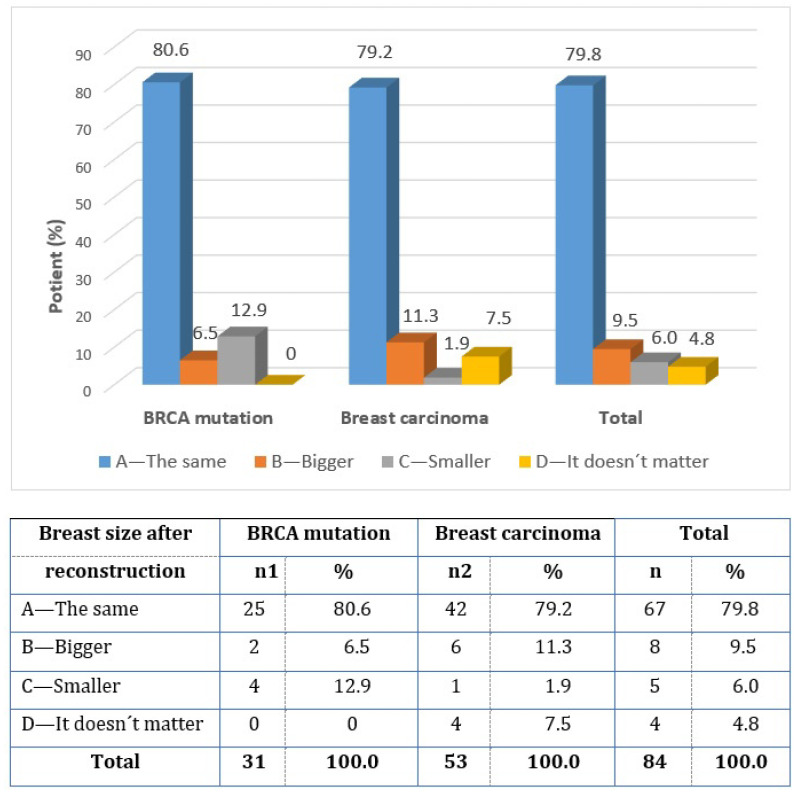
Breast size requirement after reconstruction (question 16).

All of the female respondents had knowledge and information about the different sizes and shapes of silicone implants (question 15). The opinions and wishes regarding breast size after reconstruction differed (question 16). A total of 79.8% of women chose the same breast size, 9.5% wanted bigger breasts, and 6.0% wanted smaller breasts (Figure 6). Despite possible risks, 100% of the operant women would undergo immediate breast reconstruction again (question 17).

**Figure 7:**
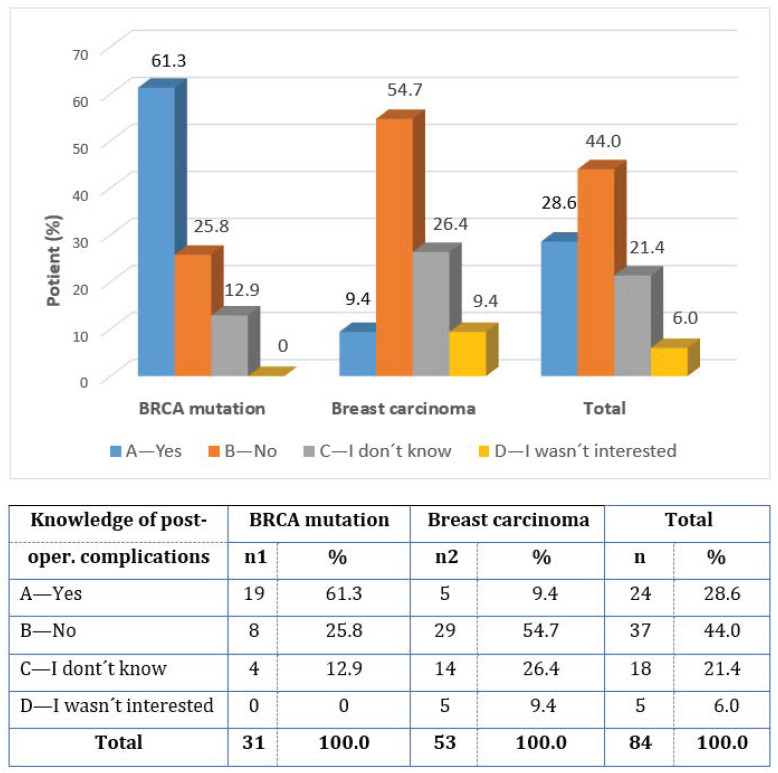
Knowledge of postoperative complications after IBR.

Almost all of the women (93%) were informed in advance about the possible operational risks, while 5% answered I DON’T KNOW, and 2% of the patients were not interested (question 18). All of the operant women knew about the possibility of postoperative complications (question 19). However, there was a fundamental difference in the awareness of the fact that postoperative complications in immediate reconstructions are more frequent than in delayed reconstructions (question 20). Only less than one-third of the female respondents knew this (28.6% of women), but the vast majority of them were younger women with BRCA mutations (61.3%) versus 9.4% of women with breast cancer. Almost two-thirds of the group did not know about this difference in advance, and 6.0% of women were not interested in it (Figure 7).

### 3.3. Evaluation of the Hypotheses of the Questionnaire Study

The results of the questionnaire study confirmed hypothesis 1, which speculates that patients with BRCA mutations are better informed about the possibility of IBR than patients with breast cancer. Conversely, hypothesis 2, which speculates that women with a genetic mutation will benefit from immediate breast reconstruction more than cancer patients, was not confirmed. The IBR was equally beneficial for both groups. Hypothesis 3, which speculates that patients with a genetic mutation will know more about possible complications of immediate breast reconstruction than patients with breast cancer, was verified and confirmed. Hypothesis 4, which speculates that patients with a genetic mutation will know more about breast reconstruction using a silicone implant than patients with breast cancer, was not confirmed.

## 4. Discussion

A prophylactic mastectomy with immediate reconstruction reduces the risk of developing malignant breast tumors in positively genetically tested women. A systematic review of twenty-one observational studies in healthy BRCA1/2 carriers describes that a risk-reducing mastectomy effectively reduces both the incidence and mortality of breast cancer [27]. Additionally, as the incidence of breast cancer increases, the demand for reconstructive procedures, especially immediate breast reconstructions, increases. The media coverage of Angelina Jolie‘s case in 2013, who underwent this procedure together with the removal of the ovaries due to BRCA 1 mutation, also contributed to increased demand for IBR. Subsequently, young women’s interest in breast cancer prevention increased in the Czech Republic and throughout the world. Health insurance companies in the Czech Republic also responded to this and started contributing to breast screening for their clients.

Immediate breast reconstruction in BRCA mutation carriers is a comprehensive set of techniques which can be used to create a breast in any patient without being dependent on an epithesis. It is thus possible to prevent later complications, possibly even disability, if the supporting skeleton is damaged. It also prevents the psychologically serious consequences of mutilation in ablation.

Before such a procedure is indicated, it is necessary to individually assess the patient’s risks via a multidisciplinary team with the participation of a clinical oncologist, general and plastic surgeon, gynecologist, clinical geneticist, and psychologist.

The incidence and availability of breast reconstruction after mastectomy varies around the world and depends on social and health status. The preferences of individual workplaces and, above all, their possibilities, are also different. As the number of breast cancer patients increases, so does the demand for reconstructive surgery. Breast reconstructions are particularly common in the USA, where half of the patients undergo reconstruction at the age of 60. Younger women undergo reconstruction more often, and older patients are usually satisfied with epithesis [15,27]. Breast replacements are also common in developed countries of Western Europe [28]. In Central and Eastern Europe, the number of reconstruction operations is lower, but there is also an increase in performance. Some women are satisfied only with the creation of a basic breast shape and do not undergo nipple and areola reconstruction [18,29].

The optimal approach to IBR with subsequent radiation therapy is not well established [30]. The opinions of breast reconstruction experts differ on whether to perform IBR or delayed reconstruction. Immediate reconstruction with skin-sparing mastectomy implants is one option for breast cancer patients undergoing neoadjuvant chemotherapy [31]. IBR using implants, followed by radiation therapy, is a safe option that can be considered in selected patients and centers [32].

Patients with breast cancer generally have a higher need for information than patients with other cancers [33]. The first sub-goal of our research was to find out how patients are informed about the possibility of immediate breast reconstruction before the operation. We assumed that patients with a genetic mutation would be more informed than patients with breast cancer. The results show that both groups of women were very well informed (100% of patients with a genetic mutation and 91% of women with breast cancer). This confirmed our first hypothesis on patients’ awareness.

Our research shows that all patients were well informed before surgery, but only just before planning the therapeutic procedure. In the vast majority of cases, the information was obtained only at a specialized workplace. We compared the results of our first hypothesis with a national study from Great Britain from 2005 to 2008, where over 18,000 women who underwent surgical treatment for breast cancer were included. Of these, immediate reconstruction was performed in 19% of patients. In our group, there were only female patients who underwent immediate reconstruction. From their entire set, 65% of patients received sufficient information about reconstructive procedures. However, 90% of patients were sufficiently informed about immediate reconstruction [34], while all patients in our group were sufficiently informed. We believe that the smaller number of patients and one operator play an important role in our favor. Several hospitals were involved in the UK study.

The sources from where the patients obtained the most information were also compared. The most detailed information was clearly provided to the patients by the plastic surgeon, who was also their operator (92% of women). The remaining 8% of patients obtained sufficient information from the Internet and from breast forums. As part of a preliminary pilot study in healthy women, it was found that almost none of the respondents knew about the possibility of immediate breast reconstruction at one time. Additionally, none knew about immediate breast reconstruction for cancer. On the other hand, almost all of the interviewed women knew about preventive mastectomy and immediate reconstruction in genetically mutated patients, which is in connection with BRCA 1 gene carriers. Nurses were mainly among the interviewees.

The second partial goal of the questionnaire study was to learn about how patients evaluate the benefits of immediate breast reconstruction. Ballard states that the availability of immediate breast reconstruction reduces the psychological trauma of a mastectomy, and it is essential to continue in this direction [16]. We believed that patients who were operated on for a genetic mutation would be more satisfied than patients with breast cancer. Breast removal is always a significant intervention in life. A woman may feel less attractive, less feminine, and feel that her body is left incomplete. Breast reconstruction for cancer is thus a good alternative by preserving femininity. This follows from our second goal, where we further investigated how important the integrity of the female body is for all interviewees.

According to Schmidt, patients who consider their breasts to be important always opt for reconstruction. Additionally, he adds that breast removal has an effect on psychosocial perception, but psychological problems can occur even after breast reconstruction [19].

We compared our results with the study described by Coufal, who states that out of 57 patients, 98% would choose the same procedure again, and 84% were overall satisfied with the reconstruction [18]. In our cohort, 100% of patients with a genetic mutation would undergo this operation again, as would all patients with breast cancer. It was not a big surprise to us that all patients evaluated the benefits of the immediate reconstruction positively. At the same time, they would recommend IBR to someone from their surroundings.

According to Durry, IBR with a free flap provides statistically higher satisfaction in a short period of time than reconstructions with silicone implants [35]. We did not deal with the comparison of operative techniques in our research. Unfortunately, as we already mentioned, not every woman is suitable for immediate reconstruction. Even some experts are in doubt about whether to perform IBR, especially in patients with cancer. Here, most plastic surgeons and breast oncologists are rather put off by reconstruction. Recently, there has been an increase in the number of immediate reconstructions at specialized workplaces.

We hypothesized that patients with a genetic mutation would know more about the possible complications of immediate breast reconstruction than patients with breast cancer. However, we found out that 61% of patients with a genetic mutation and only 9% of patients with breast cancer knew about the possible complications before surgery. We found no available study with which to compare our results.

Our fourth sub-goal was to find out whether patients know about breast reconstruction using silicone implants. We hypothesized that patients with a genetic mutation would know more about breast reconstruction using a silicone implant than patients with breast cancer. We believed that patients with a genetic mutation were younger and would be more interested in the material of the implants, their size, and possibly the brand. All the patients with a genetic mutation, as well as women with breast cancer, were well informed and aware of the use of silicone implants. Additionally, the awareness regarding possible implant sizes was excellent in both groups (100%).

At the international conference of oncology and oncoplastic breast surgery in April 2016 in Prague, Karen Flores, the Senior Breast Care Nurse Specialist from the James Paget Hospital in Great Britain, presented the very interesting and especially beneficial field of “breast care nurse”. Within this field, comprehensive care is provided to patients with breast diseases, starting with psychological support, through a detailed and comprehensible explanation of surgical treatment, to postoperative checks. Unfortunately, this beneficial field is still lacking in the Czech Republic. Its introduction into our practice must be preceded by a complex process, during which it will be necessary to adjust the education and competence of nurses so that their scope can be expanded [23].

## 5. Conclusions

Women who have breast cancer or have an increased risk of breast cancer and undergo a mastectomy should have a choice regarding whether to accept only wearing an epithesis or to undergo breast replacement surgery. The demand for reconstructive procedures is increasing, but unfortunately, not all patients undergo breast reconstruction, on the one hand, due to the lack of information about the possibilities of reconstruction, but also because of the small number of specialized centers where IBR is carried out. There is a higher risk of complications in patients with locally advanced disease. Patients with an early cancer diagnosis have significantly fewer complications. The possibility of skipping radiotherapy in patients of childbearing age with a good prognosis is essential to reduce the risk of late complications after radiotherapy (cardiomyopathy, lung fibrosis) while maintaining equally good local control of malignancy.

There are few specialized centers with a team of experts in reconstructive breast surgery in the Czech Republic, especially those that perform IBR. Our research shows that all patients were well informed about IBR, but the vast majority of them obtained the information just before the surgical procedure was planned. All women wished to maintain body integrity, and if there was a possibility of IBR, they all underwent the procedure [36].

Based on our experience supported by a questionnaire study, we recommend the following to patients: (1) talk openly about their problem; (2) obtain as much information as possible from the registering gynecologist, oncologist, or surgeon; and (3) find out more about the possibility of immediate reconstruction in specialized centers.

The recommendations for Czech healthcare management are as follows: (1) provide greater education about breast reconstruction in the clinics of gynecologists, oncologists, and surgeons; (2) interdisciplinary cooperation; (3) increase the number of centers and plastic surgeons dealing with this issue; and (4) cooperate with health insurance companies and increase the budget for the issue of breast reconstruction.

## Data Availability

The data presented in this study are available from the corresponding authors.

## Supplementary Materials

The following supporting information can be downloaded at: https://www.mdpi.com/article/10.3390/healthcare11121755/s1, Supplementary File S1: Questionnaire on women’s awareness of immediate breast reconstruction.
