# Supplementary material for: Czech Women’s Point of Views on Immediate Breast Reconstruction after Mastectomy due to BRCA Gene Mutation or Breast Cancer
Source: Healthcare (Basel). 2023 Jun 15;11(12):1755. doi: 10.3390/healthcare11121755 (PMC10298323; doi:10.3390/healthcare11121755)
Supplement: Supplementary file 1 [file healthcare-11-01755-s001.zip › healthcare-2386138-supplementary.pdf]

Questionnaire on women's awareness of immediate breast reconstruction

1. Did you undergo immediate brest reconstruction for?
A: Genetic mutation (BRCA 1,2 and others), B: Breast cancer

2. What age group do you belong to?
A: 20-29 years, B: 30-45 years, C: 46-59 years, D: 59 years and above

3. When did you learn about the possibility of immediate breast reconstruction?
A: Before diagnosis, B: During treatment planning, C: After breast removal (mastectomy), D: I don't know

4. From whom did you learn about immediate breast reconstruction?
A: General practitioner, B: Oncologist, C: Plastic surgeon, D: Serroundings, internet

5. From whom did you get the most information regarding reconstruction surgery?
A: Oncologist, B: Gynecologist, C: Plastic surgeon, D: Serroundings, internet

6. Before breast removal (mastectomy), did you know what immediate breast reconstruction means?
A: Yes, B: Rather yes, C: No, D: I don't know

7. Did you know that this procedure is covered by the health insurance company?
A: Yes, B: Rather Yes, C: No, D: I don't know

8. Do you think that there is sufficient awareness of immediate breast reconstruction in the Czech Republic?
A: Yes, B: Rather yes, C: No, D: I don't know

9. If you had to decide again, which breast reconstruction would you undergo?
A: Immediate reconstruction, B: Delayed reconstruction, C: Without reconstruction, D: I don ‘t know

10. If you underwent immediate breast reconstruction, for what reason?
A: Integrity of the body, B: Perception of the surroundings, C: I did not undergo, D: I don´t know

11. If you had someone around you with the same diagnosis, would you recommend immediate breast reconstruction?
A: Yes, B: Rather yes, C: Rather no, D: No

12. Did you know that you can perform immediate breast reconstruction with your own tissue?
A: Yes, B: No, C: I don't know, D: I wasn't interested

13. Did you know that immediate breast reconstruction with foreign material can be performed?
A: Yes, B: No, C: I don't know, D: I wasn't interested

14. Do you think that a silicone implant is harmful to the body?
A: Yes, B: No, C: I don't know, D: I wasn't interested

15. Did you know that there are different sizes and shapes of silicone implants?
A: Yes, B: No, C: I don't know, D: I wasn't interested

16. What breast size did you want to have after the reconstruction?
A: The same, B: Bigger, C: Smaller, D: It doesn't matter

17. Would you undergo immediate breast reconstruction again given the possible risks?
A: Yes, B: Rather yes, C: No, D: I don't know

18. Before the operation, were you informed about the possible risk associated with immediate breast reconstruction?
A: Yes, B: No, C: I don't know, D: I wasn't interested

19. Did you know that complications can occur after surgery?
A: Yes, B: No, C: I don't know, D: I wasn't interested

20. You know that postoperative complications with immediate reconstructions are more common than with delayed reconstructions?
A: Yes, B: No, C: I don't know, D: I wasn't interested
